# Zone-Dependent Architecture and Biochemical Composition of Decellularized Porcine Nasal Cartilage Modulate the Activity of Adipose Tissue-Derived Stem Cells in Cartilage Regeneration

**DOI:** 10.3390/ijms22189917

**Published:** 2021-09-14

**Authors:** Constanze Kuhlmann, Thilo L. Schenck, Attila Aszodi, Riccardo E. Giunta, Paul Severin Wiggenhauser

**Affiliations:** 1 Department of Hand, Plastic and Aesthetic Surgery, LMU Klinikum, University Hospital, LMU Munich, Ziemsenstrasse 5, 80336 Munich, Germany; constanze.kuhlmann@med.uni-muenchen.de (C.K.); Thilo.schenck@med.uni-muenchen.de (T.L.S.); riccardo.giunta@med.uni-muenchen.de (R.E.G.); 2 Laboratory of Cartilage Development, Diseases and Regeneration, Department for Orthopaedics and Trauma Surgery, Musculoskeletal University Center Munich (MUM), University Hospital, LMU Munich, Fraunhoferstrasse 20, 82152 Planegg, Germany; Attila.aszodi@med.uni-muenchen.de

**Keywords:** cell–matrix interaction, tissue engineering, adipose tissue-derived stem cells, ASCs, nasal septal cartilage regeneration, stem cell fate, biomimicry, extracellular matrix zone

## Abstract

Previous anatomical studies have shown different functional zones in human nasal septal cartilage (NC). These zones differ in respect to histological architecture and biochemical composition. The aim of this study was to investigate the influence of these zones on the fate of stem cells from a regenerative perspective. Therefore, decellularized porcine septal cartilage was prepared and subjected to histological assessment to demonstrate its equivalence to human cartilage. Decellularized porcine NC (DPNC) exposed distinct surfaces depending on two different histological zones: the outer surface (OS), which is equivalent to the superficial zone, and the inner surface (IS), which is equivalent to the central zone. Human adipose tissue-derived stem cells (ASCs) were isolated from the abdominal fat tissue of five female patients and were seeded on the IS and OS of DPNC, respectively. Cell seeding efficiency (CSE), vitality, proliferation, migration, the production of sulfated glycosaminoglycans (sGAG) and chondrogenic differentiation capacity were evaluated by histological staining (DAPI, Phalloidin, Live-Dead), biochemical assays (alamarBlue^®^, PicoGreen^®^, DMMB) and the quantification of gene expression (qPCR). Results show that cell vitality and CSE were not influenced by DPNC zones. ASCs, however, showed a significantly higher proliferation and elevated expression of early chondrogenic differentiation, as well as fibrocartilage markers, on the OS. On the contrary, there was a significantly higher upregulation of hypertrophy marker MMP13 (*p* < 0.0001) and GAG production (*p* = 0.0105) on the IS, whereas cell invasion into the three-dimensional DPNC was higher in comparison to the OS. We conclude that the zonal-dependent distinct architecture and composition of NC modulates activities of ASCs seeded on DPNC. These findings might be used for engineering of cartilage substitutes needed in facial reconstructive surgery that yield an equivalent histological and functional structure, such as native NC.

## 1. Introduction

The destruction of nasal septal cartilage due to trauma, skin cancer surgery, autoimmune disease or infection can lead to various problems, including impaired breathing, smelling disorders and obvious cosmetic deformity [1,2,3,4]. The surgical repair of those defects often requires costal or auricular cartilage to reconstruct nasal septal cartilage (NC). However, the harvesting of autologous cartilage bears the risk of donor site morbidity [1,5,6]. Those complications could be avoided if a viable, biocompatible and biomimetic equivalent to NC were available [7]. Hence, tissue engineering approaches aim to produce cartilage tissue satisfying surgical needs [8,9].

The most common cell sources for cartilage tissue engineering are either mature chondrocytes or adult mesenchymal stem cells (MSCs), e.g., found in adipose tissue [10,11]. The so called adipose tissue-derived stem cells (ASCs) are an ideal candidate for tissue engineering tasks [10]. Firstly, they are easily accessible through liposuction and are almost abundantly available, especially in contrast to human nasal and articular chondrocytes [12,13]. Secondly, as stem cells, they have not designated to a specific cell line yet and are able to differentiate into chondrocytes [14,15,16]. The chondrogenesis of MSCs proceeds over three intermediate steps, with a hypertrophic chondrocyte as a terminal differentiation endpoint [17,18]. Stem cells evolve via chondroprogenitors to chondroblasts and mature to chondrocytes. This complex process is accompanied by the genetic regulation of chondrogenic markers and secretion of specific extracellular matrix (ECM)-proteins, such as collagen type II and aggrecan [17,18]. ASCs are naturally found in the so-called stem cell niche of adipose tissue, where they reside in a quiescent state until they are activated by environmental cues, leading to proliferation or differentiation [19,20,21].

Artificial polymers or composite materials derived from decellularized tissue play an eminent role in current tissue engineering approaches. Recent studies also investigated xenogenic cartilage, e.g., decellularized porcine nasal septal cartilage (DPNC), as a substitute of human cartilage when decellularized ECM was chosen in the specific approach [10,22]. The decellularization process removes all native chondrocytes and pathogens, while simultaneously increasing the matrix porosity to allow for a more sufficient recellularization [23]. Decellularized cartilage has the advantage of providing a high degree of biomimicry due to its composition and three-dimensional architecture derived from native cartilage [10,24]. Previous experiments have shown that DPNC yields a proper environment for human nasal chondrocytes and ASCs that enables cartilage regeneration [23,25,26,27].

Furthermore, cell–matrix interactions play an important role in cell regulation and stem cell differentiation [28,29]. Gattazo et al. described ECM as a complex and dynamic environment that has the ability to influence and regulate cell behavior, owing to its biochemical, biophysical and biomechanical properties [30]. Biochemical communication between cells and the matrix is mediated by cell surface proteins and growth factors via direct or indirect signaling pathways [31]. The biophysical properties of ECM comprise matrix porosity, rigidity and insolubility and regulation of anchorage-related functions, such as cell division and migration [28]. The biomechanical cell–matrix interaction describes the transmission of external forces from ECM to the cell, leading to changes in cell morphology and behavior [32,33]. This process is known as mechanotransduction and is primarily conveyed by integrins [34]. A vivid example of the biophysical interaction between cells and ECM in cartilage is the circumstance that chondrocytes tend to dedifferentiate in a two-dimensional (2D) environment and start to express a spindle-shaped phenotype [35]. Dedifferentiation is accompanied by the downregulation of cartilage-specific gene expression (e.g., collagen type 2 alpha 1 (*COL2A1*) and aggrecan (*ACAN)*) and reduced ECM secretion, whereas the expression of the fibroblast marker, collagen type I (*COL1A1*), is upregulated [36]. This process could be reversed if cells are cultivated in a three-dimensional (3D) environment [35,37]. The ECM that directly surrounds a cell is described as a pericellular matrix (PCM) [38]. The PCM was identified as the main ECM compartment for the cell–matrix interaction, with the ability to specify the lineage of stem cells and contribute to resulting phenotypes [38,39,40].

Cartilage tissue architecture is intimately linked to its biochemical and biomechanical functions, which are determined by a well-balanced composition of cells and macromolecules. On the one side, there are glycosaminoglycans (GAGs) that bind water through ionic forces and lead to the typical swelling pressure of cartilage, and, on the other side, there are different types of collagens interwoven to a well described network that withstand the swelling pressure of GAGs and give support to the three-dimensional shape of the specific cartilage. Interspersed in a typical manner, chondrocytes are located within this mechanoresponsive and mechanotransducive network of the extracellular matrix. Keeping this in mind, histological evaluation of the human NC revealed three different zones within the cartilage that all possess a distinct histological architecture with a typical interplay of the extracellular matrix and cells: a superficial, intermediate and central zone [41,42]. The anatomical organization of cells and the matrix varies in between these zones. The superficial zone contains chondrocytes that express a proliferative phenotype: they are numerous, flat, small and are surrounded by dense collagen type II deposits [43]. Towards the central zone, the cell phenotype becomes more hypertrophic, spheroidal and aligned in columns within deeper layers of cartilage [41,42]. The intermediate zone appears to be a transitional zone, whereas cells of the central zone express the most differentiated phenotype, with comparably sparse collagen type II deposits in their environment. GAG and cartilage oligomeric matrix protein (COMP), as non-collagenous matrix components, are distributed more intensely and homogenously in the superficial zone, whereas they are mainly found in the direct surroundings of chondrocytes in the central zone of the nasal septal cartilage [42,43].

In this study, we hypothesize that the NC zones have distinct functions by promoting proliferation and terminal differentiation in the superficial zone and the central zone, respectively. Applying a tissue engineering approach, we suppose that ASCs that were cultured on DPNC might be influenced differently by cell–matrix interactions, depending on the specific zone the ECM was taken from. Thus, ASCs might become more proliferative and less differentiated if exposed to superficial zones when compared to ASCs exposed to central zones.

## 2. Results

### 2.1. Histological Analysis of Native Nasal Cartilage

**Figure 1:**
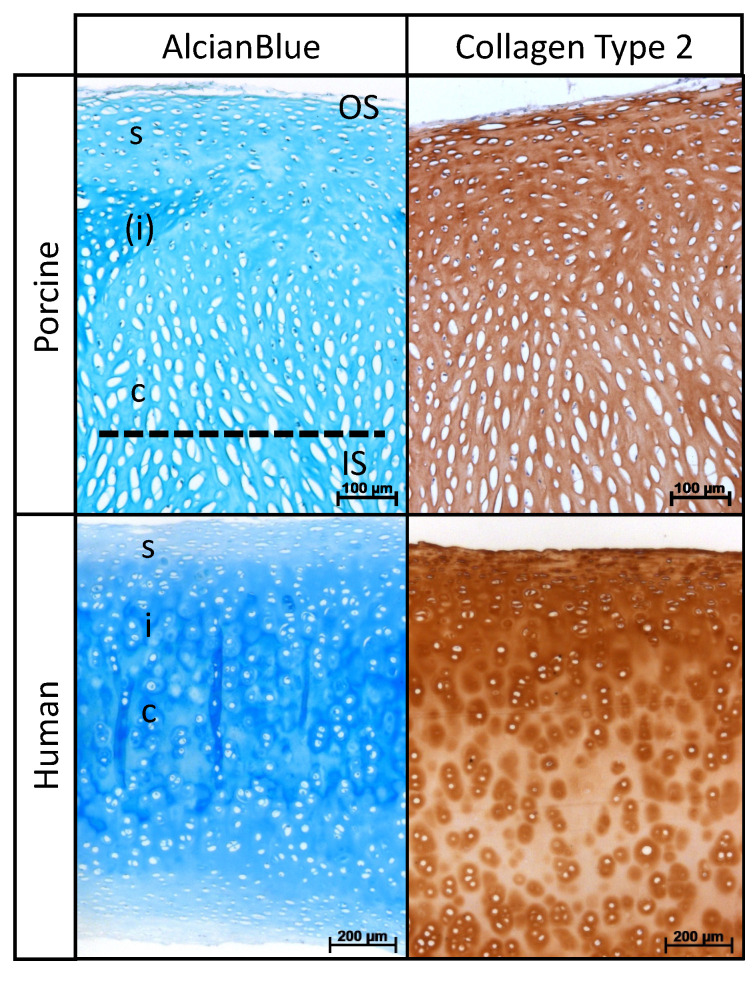
Histological organization of DPNC. Porcine and human nasal cartilage show similar zonal architecture: superficial (s), intermediate (i) and central (c) zone. Note less distinct intermediate zone in porcine cartilage marked by (i). Dotted line shows the cutting plane during preparation of scaffolds. OS identifies area of seeding on outer surface and IS on inner surface.

Histological sections of porcine and human nasal cartilage demonstrated a similar zonal architecture, according to cellular alignment (Figure 1). Alcian blue staining and collagen type II immunostaining were found to be homogenous within the entire samples. In contrast to human cartilage, a shorter intermediate zone was detectable between the superficial and central zones in porcine cartilage.

### 2.2. Evaluation of Seeding Success, Cell Distribution and Cell Vitality on DPNC Surfaces

**Figure 2:**
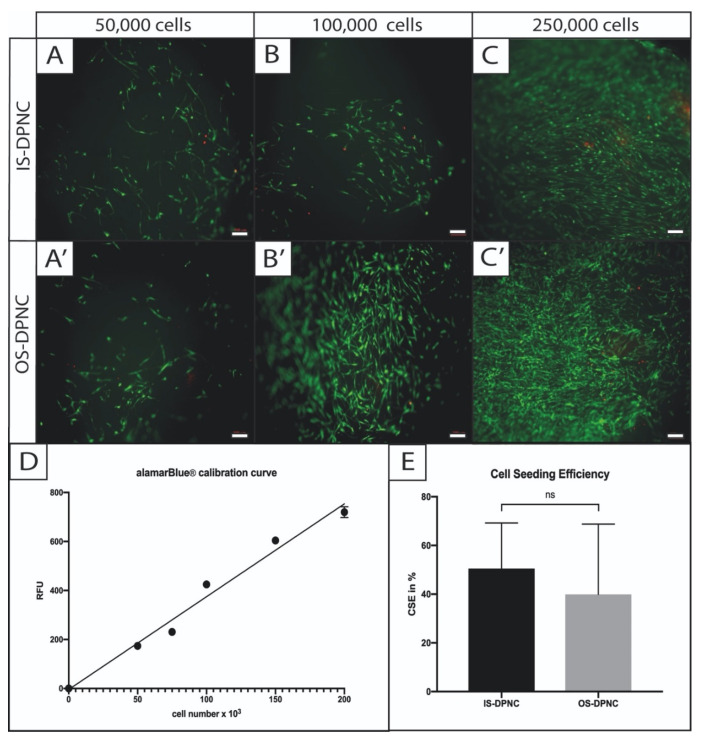
Cell seeding visualization and calculation of efficiency on different DPNC zones. ASCs were statically seeded in different concentrations on IS (**A**–**C**) or OS (**A**’–**C**’) and were visualized by Live–Dead assay 4 h later. Vital cells were stained in green (FDA) and dead cells in red (PI). No difference in cell vitality was observed between DPNC zones and cell seeding concentration (bar = 200 μm). For the calculation of cell seeding efficiency, a calibration curve (r^2^ = 0.98) was used to indirectly calculate the CSE through comparison with the MMA of defined cell numbers in 2D (**D**). CSE (**E**) revealed no significant difference (*p* = 0.3) between IS and OS (n = 5; ns = not significant; mean ± SD).

The Live–Dead (LD) assay and fluorescence imaging indicated the successful seeding of ASCs on both IS and OS surfaces of the DPNC. ASCs adhered and aligned similarly on both zones (Figure 2). There was no obvious difference regarding the cell vitality and distribution between OS and IS 24 h after seeding. The number of dead cells, represented by red PI staining, was comparably low on both scaffold surfaces. The calculated cell seeding efficiencies (CSE) were similar on both DPNC surfaces and showed no statistically significant difference (*p* = 0.511). The mean CSE of cells on IS was slightly higher, with 50.5% (±18.7% standard deviation (SD)) compared to OS (39.9 ± 28.8% SD).

### 2.3. ASC Metabolism and Proliferation

**Figure 3:**
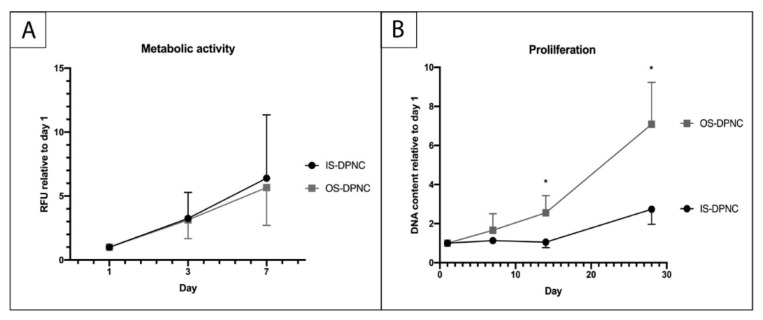
Evaluation of ASC metabolic activity and proliferation on DPNC zones. DPNC zones were seeded with 5 × 10^3^ cells and cultured for 24 h until the first time of measurement of the metabolic activity in an alamarBlue assay (**A**) and proliferation in PicoGreen assay (**B**). The relative increase in MMA of ASCs was significant over time (*p* = 0.004) and similar on both DPNC zones (n = 5). The DNA content of ASCs increased significantly on OS-DPNC in comparison to IS-DPNC after 14 and 28 days of culture, indicating that ASCs proliferate more on OS-DPNC (n = 3; * *p* < 0.05; mean ± SD).

To describe the zonal-dependent metabolic activity and proliferation capacity of ASCs, alamarBlue and PicoGreen assays were performed. ASCs exhibited a similar mitochondrial metabolic activity (MMA) in both DPNC zones. There was no significant difference regarding the aerobic aspiration between OS and IS during the first seven days of cell culture (Figure 3A). The increase in metabolic activity between days 3 and 7 was significant in both IS and OS (*p* = 0.004). PicoGreen assay monitoring proliferation showed a relative increase in DNA on both DPNC surfaces over the time period of 28 days (*p* = 0.017) (Figure 3B). The increase in DNA content was significantly higher on OS compared to IS after 14 (*p* = 0.0481) and 28 days (*p* = 0.0297).

### 2.4. Biochemical Analysis of ASC Chondrogenesis

**Figure 4:**
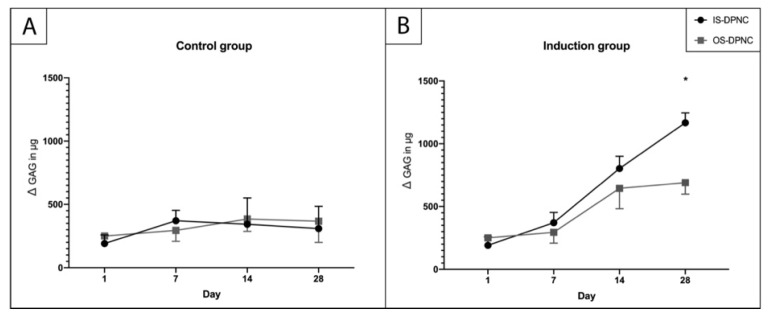
Quantification of sGAGs using DMMB assay. No increase in sGAG production occurred in control group (**A**) during the culture period. (**B**) A significantly higher (*p* = 0.0105) sGAG concentration was measured on IS compared to OS after 28 days of in vitro culture (n = 3; * *p* < 0.05; mean ± SD).

To assess the production of sulfated GAGs (sGAGs) within the scaffold zones, a 1,9-dimethylmethylene blue (DMMB) assay was performed after 1, 7, 14 and 28 days (Figure 4). There was no increase in sGAGs in the standard culture medium, neither on IS nor OS (Figure 4A), whereas a significant increase in sGAGs in the chondrogenic induction medium was observed for both surfaces over time (*p* < 0.05) (Figure 4B). Moreover, the sGAG production at 28 days was significantly higher (*p* = 0.0105) on the IS than on the OS of DPNC (Figure 4B).

### 2.5. Histological Analysis and Cell Migration

**Figure 5:**
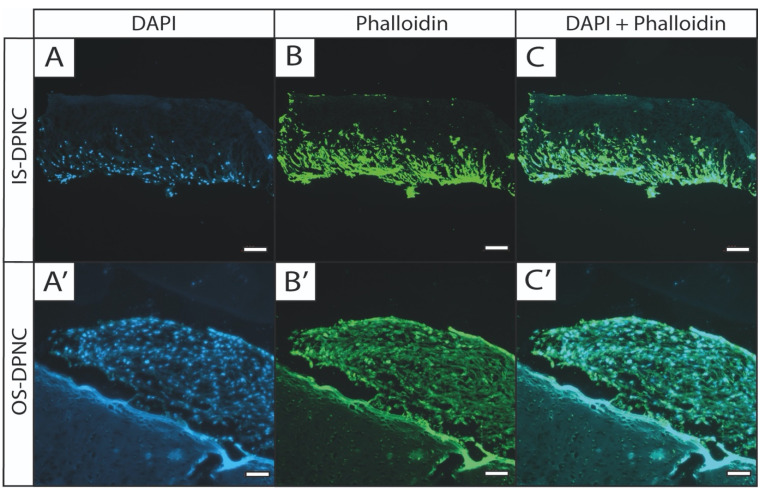
Fluorescence staining shows differences in growth pattern of ASCs between DPNC zones. DPNC scaffolds seeded with ASCs were cultured for 28 days, and were subjected to fluorescence staining with DAPI (blue, visualizes cell nucleus; **A**,**A**’) and phalloidin (green, visualizes the actin cytoskeleton; **B**,**B**’). Right column (**C**,**C**’) shows overlaid images. ASCs seeded on IS clearly migrate into the scaffold (**A**–**C**, bar = 200 μm), whereas ASCs on OS form multilayer cell stacks on surface of scaffolds (**A**’–**C**’, bar = 100 µm) (n = 3).

Phalloidin and 4′, 6-diamidino-2-phenylindole (DAPI) staining were performed to visualize cell migration through scaffolds and to observe potential morphological changes in ASCs. Cells seeded on the OS of DPNC formed stacked layers and grew around the scaffold, with only a minor infiltration into DPNC (Figure 5A’–C’). In contrast, cells seeded on IS infiltrated the scaffold throughout the central and the intermediate matrix zones after 28 days of 3D-culture (Figure 5A–C).

### 2.6. Gene Expression Analysis

**Figure 6:**
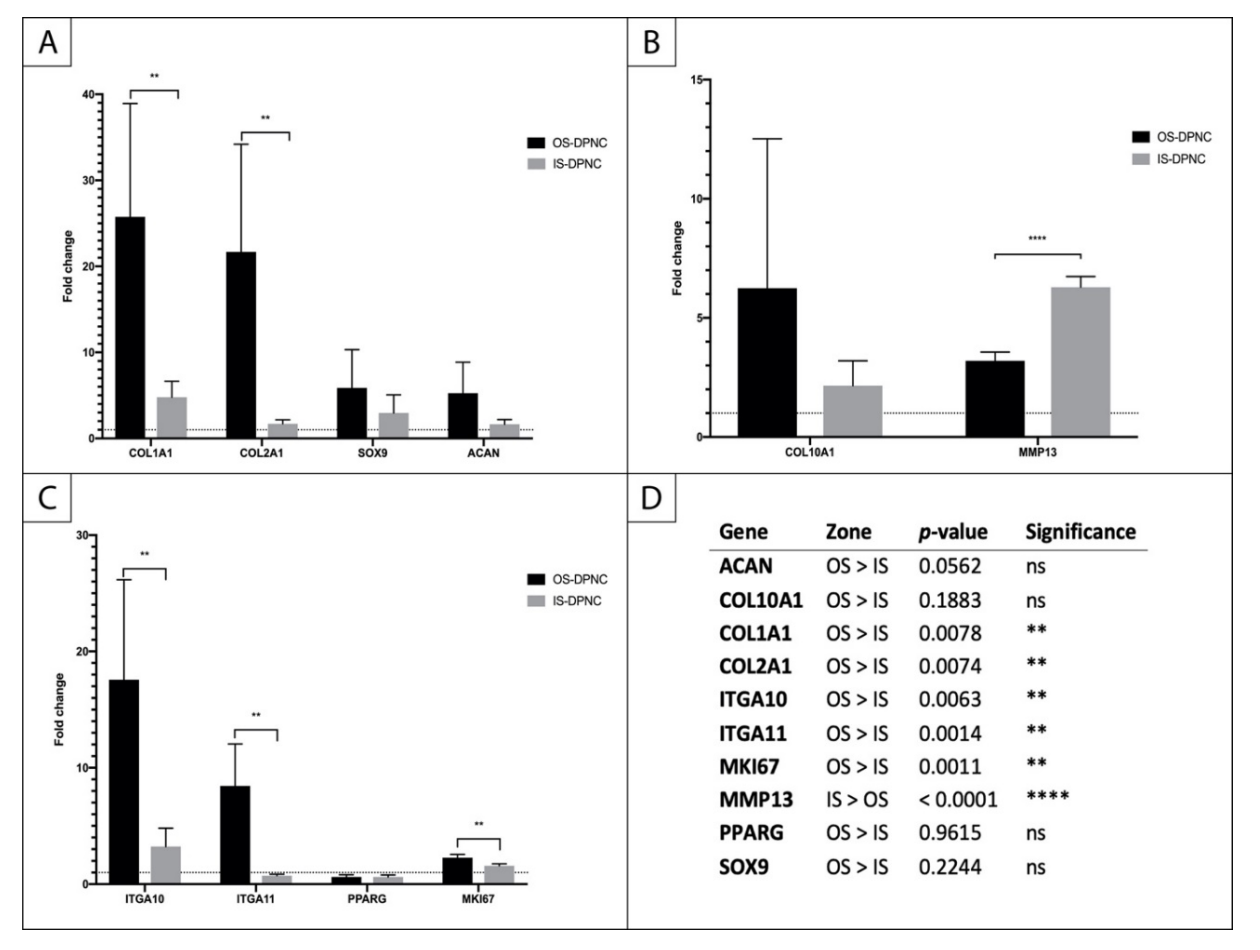
Relative changes in gene expression in ASCs seeded on IS and OS of DPNC. (**A**) early chondrogenic markers; (**B**) markers of chondrocyte hypertrophy; (**C**) integrins, adipogenesis and proliferation markers; (**D**) statistical analysis (n = 5; ** *p* < 0.01; **** *p* < 0.0001; mean ± SD).

A quantitative real-time polymerase chain reaction (qPCR) showed the expression of several chondrogenic markers in ASCs seeded and differentiated on the IS, as well as on the OS of the DPNC compared to the non-stimulated controls (Figure 6). Moreover, markers of chondrogenic differentiation were expressed differently on IS and OS. In particular, *COL2A1* was expressed almost 10-fold higher on OS compared to IS (*p* < 0.01) (Figure 6A). Similarly, SRY-related HMG-box 9 (*SOX9)*, *ACAN*, collagen type 10 (*COL10A1)* and integrin alpha 10 (*ITGA10)* showed higher levels of expression on OS (Figure 6A–C). Only matrix metalloproteinase 13 (*MMP13)* was expressed approximately 2-fold higher on IS compared to OS (*p* < 0.0001) (Figure 6B). Interestingly, the fibrocartilage/fibroblast markers *COL1A1* and *ITGA11* were also significantly upregulated in OS-seeded cells compared to IS-seeded cells (*p* < 0.01) (Figure 6A,C). The expression of the peroxisome proliferator-activated receptor gamma (*PPARG)*, a master regulator of adipocyte differentiation, is reduced in the chondrogenically-induced groups, indicating that there was no differentiation to adipose tissue. There was a significantly higher expression of proliferation marker Ki-67 (*MKI67)* on OS compared to IS (*p* = 0.0011) (Figure 6C).

## 3. Discussion

We investigated the interactions of decellularized porcine nasal cartilage with human ASCs, with a special focus on the effect of the zonal architecture of DPNC on the chondrogenic differentiation of ASCs. Previous experiments had already shown that DPNC is a conductive scaffold for human chondrocytes and is suitable for regenerating cartilage tissue. In this study, ASCs were selected as a stem cell source to investigate the influence of the cartilage ECM of the DPNC scaffold on the stem cell fate.

As the ECM provides not only a place to grow for stem cells but may also influence their cellular fate, we were looking for a suitable model to investigate the interactions of stem cells with human nasal cartilage. Several studies have previously described the zonal cellular architecture of human nasal cartilage [41,42,43]. However, it remains unclear whether this zonal organization reflects different cellular activities. It is tempting to speculate that the superficial (or peripheral) zone, with densely packed flat cells orienting parallel to the surface, represents an actively growing, proliferative area. In contrast, the central (or inner) zone, with longitudinally clustered, rounded chondrocytes, may be the site of maturation and hypertrophy, whereas the intermediate zone, with scattered oval cells, is the place where the cells switch from proliferation to differentiation. In the present study, we provided evidence that porcine nasal septal cartilage displays a zonal architecture, sGAG distribution and collagen type II distribution comparable to human septal cartilage. Thus, splitting DPNC through its central zone generates two compositionally and structurally distinct surfaces, the OS and IS, that could be used to test scaffold properties on mesenchymal stem cell behavior.

In this study, we recellularized the OS and IS surfaces of the DPNC with human ASCs and assessed their impact on cell vitality, metabolic activity and proliferation. The initial ASC vitality, MMA and CSE were not significantly influenced by the different ECM organization of OS or IS DPNC. These results demonstrate that the attachment of ASCs was comparable on both DPNC surfaces and suggest that further experiments were not affected by the seeding per se. The proliferation of ASCs were similar on both DPNC zones during the first week of the 3D culture. However, OS supported a higher proliferation rate at the second and third weeks of culture compared to IS. This observation was corroborated by the high mRNA expression level of the proliferation marker Ki-67 in ASCs seeded on OS. Histology and phalloidin staining revealed that ASCs were organized into layers on top of the OS, with only a minor infiltration into deeper zones, whereas on the IS side, the seeded cells migrated into the scaffold. As OS contains a higher density of collagen II fibrillar networks than IS, it is possible that the small pores at the OS prevent the efficient migration of the ASCs through the DPNC scaffold [43].

In our study we found that the early and midpoint chondrogenic differentiation markers, such as *COL2A1* and *ITGA10*, were upregulated to multiple levels of fold change on OS upon chondrogenic induction [44]. The early chondrogenic differentiation of mesenchymal stem cells is a hybrid situation between proliferation and differentiation. Stem cells condensate and develop the flatter and more spindle-shaped phenotype of chondroprogenitors, whereas the mRNA expression of ECM-proteins (*COL2A1*, *COL1A1*, *ACAN*) and transcription factors (*SOX9*) are upregulated [22,45,46]. Prior studies revealed that *COL1A1* is expressed in undifferentiated MSCs and that this marker indicates fibrocartilage formation or de-differentiation towards a fibroblast-like phenotype [36,47,48]. The relative expression of *COL1A1* in ASCs on OS was elevated to a 25-times fold change. Additionally, *ITGA11*, which is more expressed in dedifferentiated than in differentiated chondrocytes, was also upregulated to high levels in ASCs cultured on OS [44,49]. These transcriptional changes were accompanied by a significantly lower GAG production in comparison to cells on IS, although an increased GAG synthesis was also observed on OS after chondrogenic induction when compared with control conditions.

Taken together, these results imply that the OS of DPNC guides ASCs towards cell proliferation, early chondrogenic differentiation and fibrocartilage formation. The ASCs on OS display a similar expression pattern that has been observed in chondroprogenitors, fibrocartilage and dedifferentiated chondrocytes [36,47,48]. Chondroprogenitors are the first step in chondrogenesis and characterized as a cell population that still has the potential to undergo adipogenesis and osteogenesis, whereas these features are progressively lost in more mature chondrocytes [50,51]. However, the upregulation of the hypertrophic chondrocyte marker *COL10A1* indicates that OS-cultured ASCs are heterogenic in stem cell fate. We suggest that the cells display distinct differentiation and proliferation behavior depending on their exact location on OS. ASCs on the top of the stacked layers express fibroblast or fibrocartilage properties, whereas cells at the bottom of the stack interact with the OS ECM and are prone to chondrogenic differentiation. Further investigations of phenotypic markers expression in the OS cell layers are necessary to clarify this hypothesis. However, to create an adequate platform for cell–matrix interactions and allow for a more sufficient recellularization of the scaffold, we recommend increasing the porosity of OS for further trials.

In contrast to the appositional growth on OS, ASCs exhibited an infiltrating growth pattern if they were seeded on the IS of the DPNC. ASCs were able to migrate up to the intermediate zone of the DPNC. This invasive growth pattern was accompanied by a significantly higher sGAG production compared to OS (*p* = 0.038) upon chondrogenic induction for 28 days in a 3D culture. In accordance, mRNA levels of chondrogenic markers (*ACAN*, *SOX9*, *COL2A1* and *ITGA10*) were elevated compared to the non-stimulated control, although those markers were not as highly expressed as those in cells on OS. An earlier study revealed that the gene expression of *COL2A1* peaks at the beginning of the chondrogenesis of MSCs, and decreases during the progress of the differentiation to more advanced phenotypes; therefore, only a minor elevation of *COL2A1* is not an exclusion criteria for chondrogenesis [48]. Furthermore, *ITGA11* was not elevated in cells on IS, which indicates IS ASCs did not dedifferentiate towards fibroblast-like cells. Interestingly, the hypertrophic chondrocyte markers *COL10A* and *MMP13* were also upregulated in IS-seeded ASCs. The *MMP13* expression was significantly higher (*p* < 0.0001) on cells of IS compared to OS. Taken together, the upregulation of early and midpoint chondrogenic markers, expression of hypertrophy markers, significant sGAG production and central ECM invasion provides evidence that ASCs on IS adopt a chondrocyte behavior after 28 days of the 3D culture with the chondrogenic induction medium. As no increase in sGAG production could be observed in the control groups, the induction medium is still necessary for the chondrogenic differentiation of ASCs on any DPNC zone.

The main differences in the zones of the DPNC are the alignment of macromolecules and the three-dimensional ECM architecture [41,42]. Hypertrophic chondrocytes in IS leave large and round cavities behind after decellularization, whereas the cell niches in OS are significantly smaller, flatter, more numerous and arranged parallel to the surface [42,43]. It is well known that chondrocytes dedifferentiate in a monolayer cell culture, characterized by cell spreading and a switch from a chondrocyte gene expression profile to a fibroblastic gene expression pattern [52,53]. Zanetti et al. discovered in 1984 that inhibitors of actin polymerization reverse dedifferentiation by stimulating cell rounding, which eventually leads to redifferentiation [54]. The redifferentiation process is accompanied by the re-establishment of chondrogenic gene expression [55,56,57]. During chondrogenesis, cells condensate to spindle-shaped chondroprogenitors and mature over rounded chondroblasts to chondrocytes [57]. The round cell shape, thus, is linked to both the phenotype and differentiation status of chondrocytes [53,58,59,60] and the Rho A/ Rho-associated protein kinase (*ROCK*)-mediated actin cytoskeleton signaling pathway was identified as the mechanism behind this phenomena [61,62].

Besides 3D architecture, the DPNC also differs in ECM composition [42,43]. The different compositions of ECM ingredients are another explanation for different stem cell behavior within the zones. If the ingredients, rather than the 3D-structure of ECM zones, contribute to stem cell lineage fate, specific ECM-coated scaffolds could open new possibilities in tissue engineering. Indeed, such polymer/ECM composite grafts with a coculture of MSCs and chondrocytes showed a potential for cartilage regeneration [63]. However, Lu et al. demonstrated that regulatory changes towards the chondrogenesis of ASCs within a collagen type II scaffold were mainly induced through the downregulation of *ROCK2* gene expression, leading to a round cell shape [62]. This observation may indicate that the most important differentiation cue is the change in cell morphology through the 3D structure of ECM ingredients, and not the ingredients themselves. Nevertheless, both mechanisms are a part of the complex interaction network between cells and the matrix, and further studies are required to fully understand the sequence and nature of events that guide chondrogenic differentiation.

We consider the results of this study to be highly relevant for further practical approaches in the tissue engineering of the DPNC. The properties of the native cartilage should be seen as the gold standard in cartilage tissue engineering [7]. However, the wide majority of efforts in tissue engineering with artificial polymer compounds have focused on creating a homogeneous tissue and neglect differences in the 3D structure and composition within the cartilage. Our results indicate that the stem cell fate differs significantly between ECM zones in the DPNC, and an interaction with a specialized ECM can be used to guide ASCs towards chondrogenesis. To achieve a high degree of biomimicry, zonal-dependent differences should be considered and further investigated to provide more of an understanding of the cell–matrix interaction in order to improve the manufacturing of zonally organized, functional and native-like cartilage tissue. Based on the results of this study, a layer by layer approach that regards a zonal-dependent variation in pore size and morphology of the DPNC should be preferred in the 3D printing of artificial polymer compounds [10,64].

## 4. Material and Methods

### 4.1. Donors

Fat tissues were collected from five heathy Caucasian female donors (mean age 54.8 years ± 7.53) who underwent liposuction at the department of plastic surgery. After obtaining written informed consent, tissues were harvested with a waterjet-assisted liposuction system (Body Jet evo, human med AG, Schwerin, Germany) from the abdomen of the patients. All patients were non-smokers without significant medical history, and were tested negative for HIV and Hepatis B and C prior to surgery.

### 4.2. Cell Isolation and Culture

All tissue samples were transferred directly to the laboratory, and ASCs were isolated using 1 mg/mL collagenase (275 U/mg Collagenase Type II, Worthington Biochemical Corporation, Lakewood, NJ, USA) dissolved in Dulbecco’s Modified Eagle Medium (DMEM, Thermo Fisher Scientific, Waltham, MA, USA) in 50 mL Falcon tubes. The lipoaspirate was incubated with collagenase solution on a three-dimensional shaker (TL 10, Edmund Buehler GmbH, Bodelshausen, Germany) at 37 °C for 10 min, leading to phase separation. The upper phase was discarded and the process was repeated twice with the lower phase, which contains the stromal vascular fraction (SVF). DMEM was added to stop the enzymatic reaction, and the solution was passed through a 70 μm cell strainer (Corning, NY, USA) into a fresh 50 mL Falcon (Sarstedt AG & Co., Nuembrecht, Germany) and was centrifuged for 5 min at 500 g. The pellet was resuspended in cell culture medium (DMEM + 10% fetal bovine serum (FBS, Sigma-Aldrich, St. Louis, MO, USA), 100 U/mL penicillin and 100 µg/mL streptomycin (Life Technologies, Carlsbad, CA, USA)) and the SVF was plated in a T-175 flask (Thermo Fisher Scientific, Waltham, MA, USA) and stored in a humidified (21% O_2_, 5% CO_2_ and 37 °C) incubator (HERAcell 240i, Thermo Fisher Scientific, Waltham, MA, USA). On the next day, the flask was washed with phosphate-buffered saline (PBS, Life Technologies, Carlsbad, CA, USA) three times to remove remaining erythrocytes. The plastic-adherent ASCs were cultivated until 80% confluency, and were trypsinized, frozen and stored in liquid nitrogen until use. All ASCs were subjected to flow cytometry using positive mesenchymal stem cell markers and multilineage differentiation assays to prove their stem cell potential, as described previously [27]. The experiments were performed in triplicates, unless otherwise stated, with cells of passage 1 and 2.

### 4.3. Manufacturing and Handling of DPNC

DPNC was produced as published in the patent applications (Breiter et al., 2010) at the Institute of Bioprocess Engineering at the University of Erlangen in Germany. Briefly, the entire nasal septal cartilage was harvested from a pig belonging to the breed of Deutsches Edelschwein (Sus scrofa domestica). Cylindric discs (diameter = 5 mm, height = 1 mm) were taken from nasal septal cartilage and a chemical multistep decellularization process that included several washing steps, and treatments with NaOH H_2_O_2_, a chaotropic salt and ethanol were performed to remove all chondrocytes, cell components and pathogens [23,25]. Decellularized scaffolds were stored in a dark and dry place until further usage.

### 4.4. Histological and Immunohistochemical Analyses of Native porcine and Human Nasal Cartilage

Porcine nasal samples were acquired and prepared as previously described. Human nasal cartilage were obtained from Science Care (Phoenix, AZ, USA) as fresh-frozen anatomical specimens. All human tissue donors involved gave informed consent to Science Care. All specimens were fixed in 3.5–3.7% neutral buffered formaldehyde solution (Otto Fischar GmbH & Co. KG, Saarbrücken, Germany), embedded in paraffin, sectioned at 4 µm and stored at 56 °C overnight. Staining was then performed with acidic alcian blue solution (Otto Fischar GmbH & Co. KG, Saarbrücken, Germany), to detect sGAGs. For immunohistochemistry, deparaffinized and rehydrated sections were sequentially treated with 1% hyaluronidase (Sigma-Aldrich, St. Louis, MO, USA) in phosphate-buffered saline (PBS) and 0.2% pronase (Merck, Darmstadt, Germany) in PBS, each for 15 min at 37 °C. The collagen type II antibody (II-II6B3, Iowa City, IA, USA) was diluted 1:1000 and incubated 1 h at room temperature (RT), and the immunoreaction was detected by using the LSAB+ System-HRP (DAKO, Gloustrup, Denmark). Digital images were obtained by a Zeiss inverted light microscope (AxioObserver, Carl Zeiss, Jena, Germany).

### 4.5. Static Seeding on Different DPNC Zones

Scaffolds were sterilized and rehydrated before cell seeding as described by Schwarz et al. in a previous study [24]. The wet DPNC was lengthwise halved under sterile conditions using an anatomical forceps and a disposable scalpel (No. 20, FEATHER, Osaka, Japan). Directly after splitting the DPNC, the halves were transferred in a 24-well plate (Thermo Fisher Scientific, Waltham, MA, USA) that was coated with 0.27% agarose (Biozym LE, Hessisch Oldendorf, Germany) with either the OS or IS facing upwards. ASCs were seeded dropwise and concentrated in 50 μL medium directly on either zone. After 2 h of seeding, 1.95 mL medium was added carefully to each well. The scaffolds were cultured under the same conditions as the cells in the monolayer culture, and medium was changed three times a week.

### 4.6. Evaluation of Cell Vitality and Cell Seeding Efficiency (CSE)

4 h after seeding, a LD assay was performed to ensure seeding success, and to visualize cell distribution and vitality on different DPNC zones. For this purpose, scaffold zones were seeded directly with 5 × 10^4^, 1 × 10^5^ or 2.5 × 10^5^ ASCs and stained with LD staining solution containing 8 μg/mL fluorescein diacetate (FDA, Sigma Aldrich, St. Louis, MO, USA) and 20 μg/mL propidium iodide (PI, Sigma Aldrich, St. Louis, MO, USA) in serum-free DMEM for 5 min at 37 °C. Finally, pictures were taken with an inverted epifluorescence microscope (AxioObserver, Carl Zeiss, Jena, Germany).

CSE was calculated through the MMA of cells in a resazurin-based alamarBlue assay. Scaffold surfaces were seeded with 5 × 10^4^ cells and incubated with culture medium. After 24 h, the culture medium was replaced with alamarBlue working solution (alamarBlue cell viability reagent diluted 1:10 in DMEM, Thermo Fisher Scientific, Waltham, MA, USA) for 4 h. A total of 100 μL of each sample was transferred in a black-bottom 96-well plate (Corning, NY, USA) and the 590 nm emission wavelength was measured with a fluorescence absorbance microplate reader (Tecan SAFIRE II, Tecan Group, Maennedorf, Switzerland). AlamarBlue working solution was also added to an unseeded scaffold and was used as a blank. Relative fluorescence units (RFU) were calculated by analogously treated samples that were exposed to defined numbers of ASCs in monolayer cultures. Finally, CSE was calculated according to the following equation: CSE (%) = RFU (DPNC sample)/RFU (cell number used for seeding).

### 4.7. Influence of Matrix Zones on Metabolic Activity and Proliferation Capacity of ASCs

To evaluate the long-term influence of different DPNC zones on the MMA and cell vitality, alamarBlue assay was performed as described above at 1, 3 and 7 days after seeding. MMAs were then calculated relative to day 1 according to the following equation: RFU (day x)/RFU (day 1).

### 4.8. Biochemical Analysis of Chondrogenic Differentiation

DPNC zones (IS and OS) were seeded with 1 × 10^5^ cells and cultured either in standard medium or in chondrogenic induction medium (StemMACS ChondroDiff Medium, Milteny Biotech, Bergisch Gladbach, Germany). PicoGreen assay was performed to quantify dsDNA concentration within the scaffold zones. A DMMB assay was used to quantify sGAG production, which was calculated by division of sGAG amount by scaffold wet weight. Results were corrected for sGAG concentration of corresponding empty scaffolds, resulting in Δ sGAG. Induction was started 7 days after seeding and scaffolds were harvested at 7, 14 and 28 days, snap-frozen in liquid nitrogen and stored at −80 °C. Both assays were performed as described previously by the authors [25,27,65].

### 4.9. Histological Analyses of DPNCs

DPNCs were harvested 28 days after seeding and embedded into Tissue-Tek O.C.T. Compound (Sakura Finetec, Alphen aan den Rijn, The Netherlands) in a Tissue-Tek Cryomold^®^ (Sakura Finetec, Alphen aan den Rijn, The Netherlands) and stored at −20 °C. Fourteen-nm-thick sections were made with a cryotome (CryoStar™ NX50 Cryostat, Thermo Fisher Scientific, Waltham, MA, USA) and were stored at −20 °C until further processing. DAPI (D1306, Thermo Fisher Scientific, Waltham, MA, USA) and phalloidin (A12379, Alexa Fluor™ 488 Phalloidin, Thermo Fisher Scientific, Waltham, MA, USA) staining were used to visualize the cell nuclei and cytoskeleton, respectively. Slides were washed with 0.1% Triton X-100 (Sigma Aldrich, St. Louis, MO, USA) for 5 min and stained with DAPI for 30 s. Then the slides were washed in PBS and 50 μL of phalloidin solution (diluted 1:400 in PBS) was added dropwise on the surface and incubated in the dark for another 20 min. After washing in PBS, slides were mounted with Fluoroshield™ (Abcam, Cambridge, UK) and a cover slip. Finally, pictures were taken with an inverted epifluorescence microscope (AxioObserver, Carl Zeiss, Jena, Germany).

### 4.10. QPCR

To detect and quantify relative changes in gene expression during chondrogenic differentiation, DPNC zones (IS and OS) were seeded in duplicates with 2.5 × 10^5^ cells and cultured with chondrogenic induction medium or normal culture medium (control) for 28 days. Scaffolds were harvested in a 2 mL safe-seal Eppendorf tube (Eppendorf AG, Hamburg, Germany) and shock-frozen in liquid nitrogen. One mL Qiazol reagent (Qiagen, Venlo, The Netherlands) and a 7 mm steal bead (Qiagen, Venlo, The Netherlands) were added and the scaffolds were homogenized using mechanical lysis (Tissue Lyser LT, Qiagen, Venlo, The Netherlands) at 50 Hz for 3 × 2 min. The suspension was transferred into a fresh reaction tube and 200 µL chloroform (Sigma Aldrich, St. Louis, MO, USA) were added. Tubes were centrifuged with 12,000 rpm at 4 °C for 15 min. The clear, aqueous upper phase was transferred to a fresh tube and 70% ethanol was added in a ratio of 1:1. RNA was isolated and purified using the RNeasy Lipid Tissue Mini Kit (Qiagen, Venlo, The Netherlands) and cDNA was synthesized with the Transcriptor First Strand cDNA Synthesis Kit (Roche, Basel, Switzerland) according to the manufacturer’s instructions. Then, qPCR was performed with innuMIX qPCR DSGreen Standard qPCR Kit (Analytik Jena, Jena, Germany) in a qTower3 G Touch (Analytik Jena, Jena, Germany). Primers (Eurofins Genomics, Ebersberg, Germany) are listed in Table 1. Amplification conditions were 95 °C for 120 s, 90 °C for 30 s and 60 °C for 60 s. Data were calculated by the 2^−ΔΔCt^ method, using hypoxanthine phosphoribosyl transferase 1 (*HPRT1*) as housekeeping control gene, and were normalized to control values, which were arbitrarily set to be 1. Calculations were performed using Excel (Microsoft, Redmond, WA, USA).

### 4.11. Statistical Evaluation

Statistical analysis was performed using GraphPad Prism v8 for Mac (GraphPad Software, San Diego, CA, USA). Gaussian distribution was evaluated using a Shapiro–Wilk-test. Depending on the distribution, either an unpaired *t*-test (student’s test) or a Mann–Whitney U-test were used for statistical analysis. If more than two groups were evaluated, a two-way analysis of variance (ANOVA) approach was used. Sidak’s multiple comparisons test was used as post-hoc analyses if results were significant. Results are presented as the mean ±standard deviation (SD) and a *p*-value of <0.05 was regarded as statistically significant.

## 5. Conclusions

We have demonstrated that ASCs express different proliferation, invasion and chondrogenic differentiation behavior within different zones of the DPNC. OS significantly induced cell proliferation, but the dense collagen structure provided only minor cell invasion towards deeper layers. ASCs formed clusters on the surface and expressed a genetic upregulation of early chondrogenic differentiation and fibrocartilage markers. ASCs that were seeded on IS expressed an upregulation of chondrogenic- and hypertrophic-differentiation-related markers, such as *COL10A1* and *MMP13*, which was accompanied by a significantly higher sGAG production and higher scaffold invasion under the influence of a chondrogenic induction medium. Thus, this study provides the first evidence that the stem cell fate of ASCs can be additively guided through differences in the 3D architecture and ECM composition of nasal septal cartilage. We consider these results as highly relevant for further approaches in tissue engineering that aim to create a biomimetic, zonally-organized and native-like artificial cartilage replacement material.

## Figures and Tables

**Table 1:** Summary of target and housekeeping genes for qPCR. (nt = nucleotides, RefSeq = reference sequence).

Gene	Primer (Left)	Primer (Right)	Nt	RefSeq
Target Genes				
*ACAN*	cctccccttcacgtgtaaaa	gctccgcttctgtagtctgc	64	NM_001135.3
*COL10A1*	caccttctgcactgctcatc	ggcagcatattctcagatgga	104	NM_000493.3
*COL1A1*	ggattccctggacctaaag	ggaacacctcgctctcca	63	NM_000088.3
*COL2A1*	gtgaacctggtgtctctggtc	tttccaggttttccagcttc	94	NM_001844.4
*ITGA10*	cttttcctcgcacgtggt	gctccattccagtcataggc	70	NM_001004439.1
*ITGA11*	cttttcctcgcacgtggt	gctccattccagtcataggc	69	NM_001004439.1
*MKI67*	ccaaccaaaagaaagtctctgg	tgatggttgaggctgttcct	78	NM_001145966.1
*MMP13*	ccagtctccgaggagaaaca	aaaaacagctccgcatcaac	85	NM_002427.3
*PPARG*	tgacaggaaagacaacagacaaa	gaggactcagggtggttcag	126	NM_001330615.1
*RUNX2*	cagtgacaccatgtcagcaa	gctcacgtcgctcattttg	104	NM_001015051.3
*SOX9*	gtacccgcacttgcacaac	tctcgctctcgttcagaagtc	74	NM_000346.3
Housekeeping Gene				
*HPRT1*	tgaccttgatttattttgcatacc	cgagcaagacgttcagtcct	102	NM_000194.3
